# Inverson of the Womb

**Published:** 1859-08

**Authors:** Walter Channing


### ATLANTA MEDICAL COLLEGE.

We are informed by the Dean of the Atlanta Medical College, that the Matriculating list for the session of 1859, amounts to 166. This is an increase of one-fourth over the class of any previous year.

---

From the Boston Medical and Surgical Journal.

*Inversion of the Womb.* By WALTER CHANNING, M. D.

MESSRS. EDITORS,—You may recollect that I communicated for the JOURNAL, a few weeks since, some notes of cases of recent and chronic inversion of the womb, of which 9 were successfully treated, and 3 were fatal. Of these last, I saw neither. One was fatal one year after delivery. In neither was anything done for radical cure.

I was called to see Mrs. ——, and left town early in the morning of the 16th of the present June, and reached the address, by rail, toward evening of the same day. Her medical attendants were in waiting for me, and from them I got the following history.

Mrs. ——, aged 22, was delivered of her second child eighteen months ago. Excessive flow accompanied and followed the removal of the placenta. Inversion was discovered. No immediate attempts at reduction followed, and those afterwards made were ineffectual.—Drs. —— and —— were called to attend her some time before I met them. They did not attend her in confinement. Their attempts at relief failed. I learned further, that at the menstrual period the flow was excessive. It seemed impossible that another could be borne. Smaller losses occurred in the intervals. Mrs. —— was perfectly helpless, bloodless and emaciated. There were no symptoms of anæmia present. The blood in the superficial veins was of the natural *modena* color. Not the least pink hue was discovered. The pulse was natural in frequency, and entirely wanting in the simulative aneurismal thrill which attends true anemia. No noises or ringing of the ears, or thumping of the cerebral arteries. The heart was without complaint. It was pretty clear that something must be done to stop further loss. The back-breaking ounce was, without question, at hand. But what to do ?

Drs. —— and —— had written me before the letter of summons came, and in the mean time I had the good fortune and great pleasure to meet Prof. Peaslee in a neighboring State, and had a full talk with him about his case of reduction of a womb long inverted, and of his method of treatment. He said his case was very favorable for the operation. The walls of the abdomen were thin and very flexible, so that the hand passed readily edge-wise across from symphysis to promontory, thus giving him perfect command of the womb; not merely steadying it, but allowing him to feel its ascent should his operation be successful. There was no flow, the hand passed readily into the vagina, and complete etherization was produced. The time taken was half an hour. The first intimation he had of progress was feeling the womb pressing against the palm of his hand, which was across the brim. As soon as this was felt, he removed this hand, and almost immediately after the inverted organ regained its natural position. I asked Professor P. if he felt any progress up to the moment when the ascending womb was against his hand. "No," said he, "I was perfectly unconscious of progress till then." This is a very important fact in this most interesting history. How often has it happened to me in operative midwifery, to labor for an hour—yes, for many, and without any clear progress, when, as if in a moment, advance was obvious, and rapidly increasing, until the work was done. While writing, a case has actually occurred in which the consulting physician proposed a resort to craniotomy, as it was impossible that delivery could be effected otherwise. Almost at the moment when the suggestion was made, the head began to move, and soon was delivered.

I went to the case in —— with the important preparation derived from Prof. Peaslee. I soon found a much less favorable case than his. The walls of the abdomen were thick, tense, unyielding, at least giving no room for the hand to be passed across the brim. Then etherization was impossible. The attempt produced violent resistance with attempts to escape. The struggle produced large hæmorrhage. The state of the external organs made the passage of the hand impossible, making it difficult to reach the womb. But with all these obstacles, when I felt pressure against my fingers above the symphysis, I felt encouraged. But I soon found the bulk of the womb was in the pelvis still; that the fundus was still the lowest part, in the portion of the womb felt above the symphysis was only the anterior edge of the uninverted portion of the womb, forced up by the manipulations below. Hæmorrhage continued and increased, and it was decided to abandon the attempt at reduction and apply the ligature. This was done on the 10th of June. The womb came away on the 27th, seventeen days after the operation—thirteen days sooner than happened in my second case. Dr. —— writes on the 27th, "It (the instrument) came away on the seventeenth day from its application. The patient is doing well—*very well.* At one time considerable vomiting and sharpness of face, &c. There is now appetite, and improving strength."

This case, added to those before communicated, makes the whole number thirteen, ten of which were successfully treated.

*July 1st,* 1859.

